# Cytokine release syndrome and cancer immunotherapies – historical challenges and promising futures

**DOI:** 10.3389/fimmu.2023.1190379

**Published:** 2023-05-25

**Authors:** Deep Shah, Brian Soper, Lindsay Shopland

**Affiliations:** ^1^ In vivo Services, The Jackson Laboratory, Sacramento, CA, United States; ^2^ Technical Information Services, The Jackson Laboratory, Bar Harbor, ME, United States

**Keywords:** cancer immunotherapy, cytokine release syndrome, CAR-T cells, bispecific T cell engagers, immune checkpoint inhibitors

## Abstract

Cancer is the leading cause of death worldwide. Cancer immunotherapy involves reinvigorating the patient’s own immune system to fight against cancer. While novel approaches like Chimeric Antigen Receptor (CAR) T cells, bispecific T cell engagers, and immune checkpoint inhibitors have shown promising efficacy, Cytokine Release Syndrome (CRS) is a serious adverse effect and remains a major concern. CRS is a phenomenon of immune hyperactivation that results in excessive cytokine secretion, and if left unchecked, it may lead to multi-organ failure and death. Here we review the pathophysiology of CRS, its occurrence and management in the context of cancer immunotherapy, and the screening approaches that can be used to assess CRS and de-risk drug discovery earlier in the clinical setting with more predictive pre-clinical data. Furthermore, the review also sheds light on the potential immunotherapeutic approaches that can be used to overcome CRS associated with T cell activation.

## 1 Introduction

According to the World Health Organization, cancer is the leading cause of death worldwide, accounting for 10 million deaths in 2020 i.e., one in six deaths. In the United States (U.S.) alone, the American Cancer Society estimates that there will be a little over 1.9 million new cases and 609,820 deaths in 2023. In addition to traditional cancer targeting approaches like radiation, chemotherapy and small molecules that interfere with the cancer signaling pathways, immunotherapies that harness the patient’s immune system to fight cancer are revolutionizing cancer therapy.

Normally the innate and the adaptive immune system works in a coordinated fashion to eliminate the cancer cells. Cancer arises when the tumor cells evade the host’s immune system. However, new therapies that enhance the immune system to eliminate cancer cells have significantly improved survival for a number of indications, including hematologic malignancies and a subset of solid tumors. Notably, certain types of these so-called “immunotherapies” can also lead to potentially fatal adverse reactions. Chief among these is Cytokine Release Syndrome (CRS), a systemic inflammatory response that is caused by the large, rapid release of cytokines in the blood by immune cells which may result in multi-organ failure and death (1). The term “cytokine release syndrome” was first described in early ‘90s when anti-T cell antibody Muromonab – CD3 (OKT3) was used clinically as an immunosuppressant for organ transplantation (2). Since then, CRS has been reported in the context of influenza infections (3), COVID-19 caused by severe acute respiratory syndrome coronavirus -2 (SARS-CoV-2) (4), graft versus host disease (5), hematopoietic stem cell transplantation (6), and during the use of cancer targeted immunotherapies such as immune checkpoint inhibitors (7), bispecific T cell engagers (8), and Chimeric Antigen Receptor (CAR)–T cells (9).

The current review will focus primarily on CRS associated with the predominant classes of cancer immunotherapies. Following a review of the basic pathophysiology of CRS, the mechanism of each major class of cancer immunotherapy will be described. The relative CRS incidence is higher for CAR-T therapy as compared to bispecific T cell engagers (10), and reports have emerged about CRS incidence post immune checkpoint inhibitor therapy as well (7, 11–14). The review will shed light on CRS incidences with current FDA approved therapies and the CRS management. Finally, a summary of strategies being developed to prevent or reduce CRS will be presented.

## 2 Pathophysiology of CRS

As the name implies, CRS is clinically manifested when the inflammatory cytokines are released by activated lymphocytes (T cells, B cells, and NK cells) and/or myeloid cells (monocytes, macrophages, and dendritic cells) or by non-immune cells, such as endothelial cells. It is a non-antigenic toxicity related to the hyperactivation of the immune system. The term CRS has been used interchangeably with “Cytokine Storm” although cytokine storm was coined in reference to immune system activation independent of tumor targeting. CRS is more specifically used in the context of cancer immunotherapy (15, 16).

The cytokine profile varies between diseases i.e., the cytokines triggered during a viral infection are different from the ones released during cancer immunotherapy (17). However, the symptoms of CRS across diseases have common features and generally result in organ/system damage and lung, liver, or kidney dysfunction.

When the pattern recognition receptors (PRRs) on the membrane of antigen presenting cells (APCs) bind to damage associated molecular patterns (DAMPs) or pathogen associated molecular patterns (PAMPs), immune cells and epithelial cells are stimulated to release cytokines. This response results in recruitment and activation of the innate immune cells, including macrophages, neutrophils, and NK cells. Interleukin-1 (IL-1), Interleukin-6 (IL-6), Tumor Necrosis Factor-alpha (TNF-α), and Interferon-gamma (IFN-γ) are critical to cytokine storm/CRS resulting from infection/immunotherapy respectively. The review by Peixian Chen et al. illustrates the downstream signaling pathways activated by the critical cytokines. The innate immune cells activate each other and release cytokines to further activate the adaptive immune system’s T cells and B cells. Continuous exposure to external stimuli overwhelms the counter-stimulatory negative feedback loop that is responsible for restricting the inflammatory damage and leads to a positive feedback loop whereby the activated adaptive immune cells act on the innate immune cells and upregulate the cytokine levels. Thus, there is an accumulation of the immune cells in local tissues and extensive production of cytokines which results in cytokine storm and overactivation of the immune system. The immune cells and the released cytokines induce endothelial dysfunction, capillary leakage, and pyroptosis resulting in multiple organ damage and function failure (18). The specific mechanisms by which cancer immunotherapies induce CRS are therapy dependent.

## 3 CAR-T cells

In CAR-T therapy, the T cells isolated from the patient’s blood through leukapheresis are engineered to express the chimeric antigen receptor, expanded, and infused back into the patient. The CAR expressing T cells attach to a specific antigen on cancer cells in an MHC (Major Histocompatibility Complex) independent fashion and are better equipped to specifically target the tumor.

**Figure 1:**
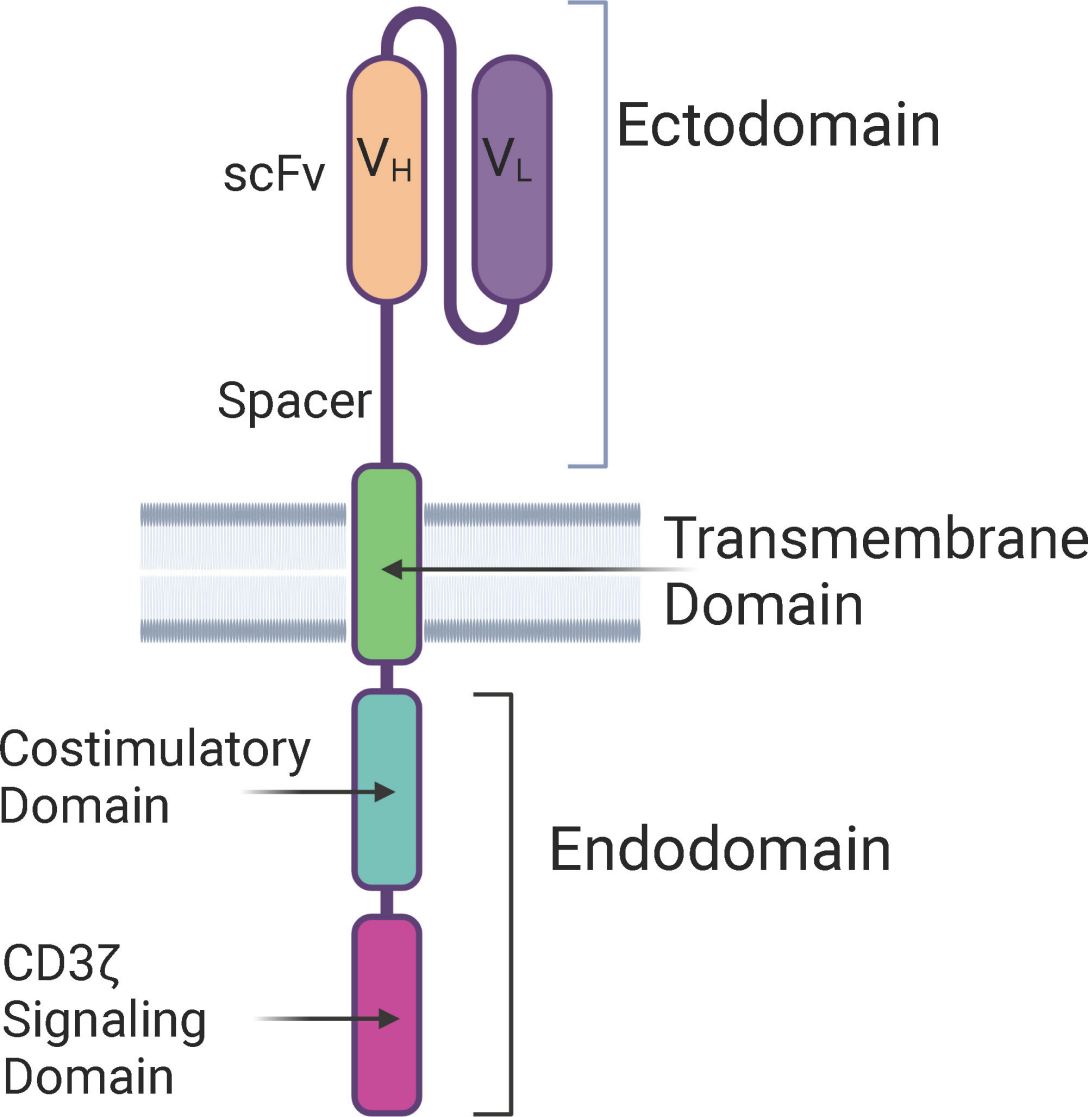
Structure of a Chimeric Antigen Receptor (CAR) – The basic components of a CAR consists of an extra-cellular ectodomain, a transmembrane domain, and an intra-cellular endodomain. CAR-T cells can be categorized into five generations based on the modifications in the endodomain, including type and number of costimulatory domains, transgene for cytokine secretion, and the IL-2 receptor intracellular domain. scFv, Single-chain variable fragment; V_H_, Heavy chain variable region; V_L_, Light chain variable region.

The structure of CAR-T cells consists of an ectodomain, a transmembrane domain, and an endodomain. The ectodomain is exposed to the extracellular space and consists of a signal peptide, antigen recognition site, and a spacer. The transmembrane domain consists of a hydrophobic alpha helix that spans the membrane and serves as an anchor to the T-cell membrane. The intracellular endodomain is the functional part of the receptor and initiates the signaling cascade that activates the T cell. It consists of an intracellular CD3 ζ signaling domain and a costimulatory domain for full T cell activation (**Figure 1**) (19).

Based on the intracellular signaling domain, CAR-T cells can be categorized into five different generations:

First-generation CAR-T cells contained a single CD3 ζ chain signaling domain without any costimulatory domains. For this generation, the cells had low IL-2 production and had to be supplemented with IL-2. Also, the CAR-T cells had low proliferation and short *in vivo* lifespan (19–21).

Second generation CAR-T cells contained a costimulatory motif (CD28 (22), OX-40 (23), or 4-1BB (24)) in addition to the CD3 ζ chain signaling domain to drive cytokine production and T cell proliferation. Studies showed that 4-1BB ζ-CAR-T cells persist longer than CD28 ζ-CAR-T cells and ameliorate T-cell exhaustion induced by tonic signaling of CARs. CD28 ζ-CAR-T cells in the context of some CARs could lead to constitutive activation of T cells in the absence of an antigen and can have poor antitumor effects (25–28). CAR-T cells containing the OX-40 signaling domain were found to have more enhanced proliferation and immune memory compared to 4-1BB CAR-T cells under repeated stimulation with BCMA-expressing target cells. This evidence supports their potential use in patients with relapsed multiple myeloma (29). In a screen with different costimulatory receptors, a CAR with antigen-independent OX-40 signaling was found to be most effective for the treatment of leukemia and metastatic lymphoma (30). It is critical to note that optimal CAR design is highly disease and target dependent.

Third generation CAR-T cells contained multiple costimulatory domains: CD3ζ-CD28-OX-40 or CD3ζ-CD28-41BB. Based on initial reports, the idea was that the presence of 4-1BB/OX-40 would allow the CAR-T cells to persist longer, and CD28 would cause rapid tumor elimination. The third generation CAR-T cells produce cytokine, proliferate, persist longer, have a better safety profile, and can be valuable for the treatment of patients with low disease burden/minimal residual disease (31, 32). However, some studies reported no difference in cytokine production and anti-tumor activity (33) or worse performance by third generation CAR-T as compared to the second generation (34, 35), possibly due to the overstimulation of CAR-T leading to exhaustion.

Fourth generation CAR-T cells are designed based on the second generation of CAR-T cells and contain an additional transgene for constitutive or inducible cytokine secretion, specifically IL-7, IL-12, IL-15, IL-18, or IL-23. They are known as TRUCKs i.e., T cells redirected for universal cytokine-mediated killing. TRUCK CAR-T cells demonstrate enhanced effector function and durability of effector cells. In the pre-clinical setting, the presence of cytokine greatly enhanced the efficacy of CAR-T cell therapies and eliminated systemic toxicity because the cytokines are directly deposited in the target tissue through CAR induced release (36–39).

Fifth generation CAR-T cells are also based on the second generation and contain an additional intracellular domain of the IL-2 receptor that allows antigen dependent activation of the JAK/STAT pathway. This causes CAR-T cells to generate memory T cells with the potential for a more durable long-term response (40, 41). Fifth generation CAR-T cells aim to address the CRS toxicity by promoting local effector cytokine production and stimulation upon contact with the tumor antigen and preventing excessive cytokine production that causes CRS (42).

Besides these five generations of CAR-T cells, other approaches to enhance CAR-T cells recognition and activation include –

➢ Dual CAR-T cells for targeting two antigens that are co-expressed on the cancer cell. This would enhance the efficacy when both antigens are engaged with the CARs (43).
➢ Split CAR-T cells where the CD3 ζ chain signaling domain and the costimulatory CD28/4-1BB are located on two different CARs. Simultaneous engagement of the two CARs with two different antigens would lead to full T cell activation. Both of these approaches have shown promising results in the pre-clinical setting and are currently being investigated in multiple clinical trials (44).
➢ CAR-T cells with engineered costimulation for providing integrated CD28 and 4-1BB signals. CD28 and CD3 ζ signaling domains, along with the 4-1BB ligand, would provide optimal costimulatory support to enhance the anti-tumor efficacy and prolong the persistence of CAR-T cells (45).

Strategies have also been proposed to minimize the activation and unwanted off tumor effects of CAR-T cells. These include –

➢ Drug inducible CARs where the recognition and signaling domain are on different polypeptides and contain a drug-inducible heterodimerization domain (46).
➢ Universal CARs with fragment crystallizable (Fc) receptor domain that can bind to an adaptor antibody to target the tumor associated antigen. Universal CARs minimize off tumor adverse effects by controlling the activity of CAR-T based on the half-life of the adaptor antibody (47).
➢ Inhibitory CARs (iCARs) where the T cell expresses two CARs – one that contains a stimulatory and costimulatory domain and binds the tumor specific antigen, and the other one that is linked to an inhibitory domain (Programmed cell death protein -1 (PD-1)/Cytotoxic T-Lymphocyte Associated Protein 4 (CTLA-4)) and specific to an antigen expressed on the normal healthy cells. This approach would help to better distinguish between healthy and cancerous cells and would provide a better safety profile (48).
➢ Biotin based CARs called Biotin Binding Immune Receptors (BBIR) where extracellular-modified dimeric avidin is linked to the intracellular T-cell signaling domain. BBIR T cells selectively bind to the cancer cells that are pretargeted with specific biotinylated molecules. Multiple antigens can be targeted simultaneously or in a sequential manner using this approach (49). Affinity enhanced biotin binding CAR-T cells include monomeric streptavidin instead of dimeric avidin in the CAR system (50).
➢ Inducible apoptosis using an inducible caspase9 (iCasp9) cell suicide system, which allows for the removal of inappropriately activated CAR-T cells. It acts as a safety switch to eliminate the cells during on-target, off-tumor activity induced toxicity. It is based on the fusion of human caspase 9 to a modified human FK-binding protein, allowing conditional dimerization. A drug called AP1903 is administered during an adverse event which causes dimerization and activation of iCasp9, resulting in the induction of apoptosis and removal of the CAR-T cells expressing high levels of transgene (51, 52).

Even though the development of CAR-T cells has been ongoing for almost three decades [16], the first CAR-T therapy targeting CD19 antigen on cancer cells, Kymriah, was approved by the U.S. Food and Drug Administration (FDA) in 2017 for the treatment of children or young adults with B cell precursor Acute Lymphoblastic Leukemia (ALL). Since then, five additional CAR-T therapies have been approved by the U.S. FDA for various hematologic malignancies including leukemias, lymphomas, and multiple myeloma (53). **Table 1** highlights the FDA approved CAR-T cell therapies and the reported CRS incidence.

**Table 1:** US-FDA approved CAR-T Therapies.

Generic Name	Brand Name	Target Antigen	CAR-T Generation and Costimulatory Domain	Targeted Disease	CRS Manifestation	%Incidence
Tisagenlecleucel	Kymriah	CD19	2nd Gen (4-1BB)	Pediatric and Young Adult with relapsed or refractory B-cell acute lymphoblastic leukemia (ALL)	Fever, hypotension, hypoxia, tachycardia, and may be associated with hepatic, renal, and cardiac dysfunction, and coagulopathy.	77% of patients with relapsed or refractory ALL including ≥ grade 3 (Penn Grading System) in 48% of patients
				Adult patients with relapsed or refractrory Diffuse large B cell lymphoma (DLBCL)		74% of the patients with r/r DLBCL including ≥ grade 3 (Penn Grading System) in 23% of patients
				Adult patients with relapsed or refractory (r/r) follicular lymphoma (FL)		53% of the adult patients with r/r FL
Axicabtagene ciloleucel	Yescarta	CD19	2nd Gen (CD28)	Adult patients with relapsed or refractory large B cell lymphoma	Fever, hypotension, tachycardia, chills, hypoxia, headache, and fatigue. Serious events that may be associated with CRS include, cardiac arrhythmias (including atrial fibrillation and ventricular tachycardia), renal insufficiency, cardiac failure, respiratory failure, cardiac arrest, capillary leak syndrome, multi-organ failure, and hemophagocytic lymphohistiocytosis/macrophage activation syndrome	93% of patients with large B-cell lymphoma (LBCL), including ≥ Grade 3 CRS in 9%
				Adult patients with relapsed or refractory (r/r) follicular lymphoma (FL)		84% of patients with r/r FL including ≥ Grade 3 CRS in 8%
Brexucabtagene autoleucel	Tecartus	CD19	2nd Gen (CD28)	Adults with relapsed or refractory Mantle Cell Lymphoma	Fever, hypotension, tachycardia, chills, hypoxia, headache, fatigue, and nausea. Serious events associated with CRS in MCL and ALL included hypotension, fever, hypoxia, tachycardia, and dyspnea.	91% of patients with MCL, including ≥ Grade 3 (Lee grading system1) CRS in 18% of patients.
				Adult with relapsed or refractory B-cell acute lymphoblastic leukemia (ALL)		92% of patients with ALL, including ≥ Grade 3 (Lee grading system1) CRS in 26% of patients.
Lisocabtagene maraleucel	Breyanzi	CD19	2nd Gen (4-1BB)	Adult patients with relapsed or refractory large B cell lymphoma	Fever, hypotension, tachycardia, chills, hypoxia, and headache. Serious events that may be associated with CRS include cardiac arrhythmias (including atrial fibrillation and ventricular tachycardia), cardiac arrest, cardiac failure, diffuse alveolar damage, renal insufficiency, capillary leak syndrome, hypotension, hypoxia, and hemophagocytic lymphohistiocytosis/macrophage activation syndrome	46% of patients with large B cell lymphoma, including ≥ Grade 3 CRS (Lee grading system1) in 3.1% of patients
Idecabtagene vicleucel	Abecma	BCMA	2nd Gen (4-1BB)	Adult patients with relapsed or refractory Multiple myeloma	Pyrexia, hypotension, tachycardia, chills, hypoxia, fatigue, and headache. Grade 3 or higher events that may be associated with CRS include hypotension, hypoxia, hyperbilirubinemia, hypofibrinogenemia, ARDS, atrial fibrillation, hepatocellular injury, metabolic acidosis, pulmonary edema, multiple organ dysfunction syndrome and hemophagocytic lymphohistiocytosis/macrophage activation syndrome (HLH/MAS)	85% of patients with multiple myeloma. Grade 3 or higher CRS (Lee grading system1) occurred in 9% of patients, with Grade 5 CRS reported in one (0.8%) patient.
Ciltacabtagene autoleucel	Carvykti	BCMA	2nd Gen (4-1BB)	Adult patients with relapsed or refractory Multiple myeloma	Pyrexia, hypotension, increased aspartate aminotransferase (AST), chills, increased alanine aminotransferase and sinus tachycardia. Grade 3 or higher events associated with CRS included increased AST and ALT, hyperbilirubinemia, hypotension, pyrexia, hypoxia, respiratory failure, acute kidney injury, disseminated intravascular coagulation, HLH/MAS, angina pectoris, supraventricular and ventricular tachycardia, malaise, myalgias, increased-Creactive protein, ferritin, blood alkaline phosphatase and gamma-glutamyl transferase	95% of patients with multiple myeloma. Grade 3 or higher CRS (2019 American Society of Transplantation and Cellular Therapy - ASTCT grade)1 occurred in 5% of patients, with Grade 5 CRS reported in 1 patient.

The information is based on the package insert of each FDA approved CAR-T therapy.

CAR-T associated CRS is manifested by the cytokines released upon the administration of T cells or upon the activation and release of cytokines by other immune cells in response to CAR-T activation. In their reviews, Wei et al. (54) and Morris et al. (55) have described the model for the occurrence and evolution of CRS upon CAR-T administration: Upon infusion, the CAR-T cells recognize and bind to the tumor-specific antigen, which results in the activation and proliferation of CAR-T cells at the tumor site. Cytokines and inflammatory mediators (interferon (IFN)-γ, tumor necrosis factor (TNF)-α, granulocyte macrophage colony stimulating factor (GM-CSF), and catecholamines) are released by activated CAR-T cells and the tumor microenvironment which induces tumor killing and leads to the initiation of a cascade of cytokine release (about 0-5 days post-CAR-T infusion). A local inflammatory response is triggered which is enhanced by tumor infiltrating macrophages and dendritic cells. The pattern recognition receptors (PRRs) on the macrophages identify the damage associated molecular patterns (DAMPs) on the pyroptotic tumor cells and enhance the activation of monocytes/macrophages in a contact dependent fashion through CD40 (expressed on macrophage)-CD40L (expressed on CAR-T cell). IL-6, IL-1, and nitric oxide (NO) are released by activated macrophages which drives the systemic CRS response. There is a proliferation of CAR-T cells and an increase in the levels of cytokines in the peripheral blood. The systemic response causes endothelial damage and vascular leakage into several organs which can lead to organ failure. The levels of cytokines and CAR-T cells in the peripheral blood continue to rise until they peak (about 1-2 weeks post infusion). T cells, cytokines, and activated monocytes migrate into the nervous system following the breakdown of the blood brain barrier and can lead to an immune cell associated neurotoxic syndrome (ICANS). In the final phase (about three weeks post CAR-T infusion), tumor eradication leads to a decrease in antigen stimulation, and therefore the number CAR-T cells and cytokines decline in the peripheral blood (54–59).

Clinical trial NCT03919240 investigated the impact of tumor burden on CRS severity and efficacy of CD19 CAR-T cells on relapsed/refractory B-ALL. The results of the study demonstrated that the patients with lower tumor burden had lower CRS severity and better efficacy in terms of complete remission as compared to the patients with higher tumor burden (60). Besides this trial, several other studies have assessed the factors/biomarkers associated with occurrence of severe CRS which include pre-treatment tumor burden, CAR-T cell dose, CD4/CD8 CAR-T cell ratio, peak CAR-T cell *in vivo* expansion, and the measurement of serum cytokine levels at specific timepoints post-CAR-T infusion (61, 62). Thus far, any correlations of these factors with CRS severity are context dependent.

CAR-T therapies for the treatment of solid tumors have been challenging due to the presence of tumor heterogeneity. As compared to hematologic malignancies, solid tumors tend to express multiple antigens, and the antigens are present on normal cells leading to higher chances for “on-target off-tumor” toxicity. Antigen loss and antigen escape have been seen in clinical trials with CD19 CARs for treatment of ALL. These concerns are even greater for solid tumors due to the presence of multiple antigens. For this reason, use of dual antigen CARs has been shown to be more effective in solid tumors (63, 64).

The tumor microenvironment is a further complicating factor. The penetration of CAR-T cells in solid tumors is restricted due to inhibitory tumor vasculature and dense fibrogenic extracellular matrix. Vascular endothelial growth factor (VEGF) promotes tumor growth, inhibits the maturation of dendritic cells, and activates Tregs and myeloid-derived suppressor cells (MDSCs). Also, it inhibits the infiltration of T cells by suppressing intracellular adhesion molecule (ICAM-1). Using a VEGF inhibitor, Bevacizumab, along with the CAR-T cells enhanced the tumor infiltration and anti-tumor efficacy in a preclinical model of human neuroblastoma (65, 66).

In addition to the aberrant tumor vasculature, altered metabolism and cancer cell proliferation results in hypoxic tumor microenvironment which is prone to oxidative stress. The tumor microenvironment also contains cancer associated fibroblasts (CAFs), tumor associated macrophages (TAMs), MDSCs, Tregs, and tumor associated neutrophils (TANs) that suppress the T cell immune response. Moreover, tumor cells express Gal9 and Programmed death-ligand 1 (PD-L1) that bind to the T cell inhibitory receptors T cell immunoglobulin and mucin domain 3 (TIM-3) and PD-1, respectively, which lead to T cell exhaustion. Combination therapy of CAR-T cells with checkpoint inhibitors and using TRUCKs to combat the immunosuppressive microenvironment by secreting cytokines locally serve as great strategies to overcome this challenge (67–69). Collectively, the complexities associated with solid tumors have limited tumor responses to CAR-T and have also therefore led to a gap in data associated with cytokine release mediated toxicities.

CRS management for CAR-T therapy is a critical patient need, and the type of intervention depends on the severity of CRS. The American Society of Clinical Oncology (ASCO) released updated guidelines for the management of immune-related adverse events (irAEs) in patients treated with CAR-T using a grade-based system. CRS grading is based on the consensus criteria developed by the American Society of Transplantation and Cellular Therapy (ASTCT) in 2019.

Grade 1 is indicated by fever greater than or equal to 38°C without hypotension or hypoxia. The management includes supportive care with antipyretics, hydration, and broad-spectrum antibiotics in case of an infection.

Grade 2 includes hypotension that does not require vasopressors and/or hypoxia requiring low flow oxygen in addition to fever. Management for Grade 2 includes supplemental oxygen and treatment with the IL-6 inhibitor Tocilizumab in addition to supportive care outlined for Grade 1. Corticosteroids are suggested for prolonged, refractory CRS.

Grade 3 CRS is characterized by fever, hypotension requiring vasopressors, and/or hypoxia requiring high flow oxygen. For Grade 3 CRS, the patient is admitted to the intensive care unit, and an echocardiogram is performed to assess the cardiac function in addition to care outlined for Grade 2 CRS.

Grade 4 CRS is associated with fever, hypotension requiring multiple vasopressors, and/or hypoxia requiring positive pressure. In addition to the care outlined for Grade 3 CRS, mechanical ventilation is provided, as needed.

Grade 5 CRS is defined as death due to CRS where another cause is not the main factor of death (70, 71).

Elevated IL-6 in patients receiving CAR-T therapy is associated with severe CRS. So, CRS management primarily includes the IL-6 receptor antagonist, Tocilizumab. It is the FDA approved standard of care and has led to the reversal of CAR-T associated CRS (72). Corticosteroids help to suppress the inflammatory response. Corticosteroids are promptly administered if there is no improvement in symptoms post Tocilizumab treatment. Possible mechanisms for non-response/insufficient response to Tocilizumab could be inadequate dosing, the timing of tocilizumab administration, the role of alternate cytokines driving CRS, or a compensatory feedback loop in IL-6 signaling (73–75). Siltuximab is an IL-6 antagonist that prevents the binding of IL-6 with the soluble form of the IL-6 receptor as well as the membrane bound receptor. Several studies indicate that it could be used either alone or in combination with Tocilizumab to combat refractory cases of CRS. A clinical trial (NCT04975555) is underway to investigate the role of Siltuximab in the treatment of CRS and ICANS related to CAR-T cell therapy.

Notably, a recent meta-analysis that involved 2592 patients across 84 studies found that all-grade CRS rate and ≥ 3 CRS rate was significantly higher in hematologic malignancies (all-grade: 81%; grade ≥ 3: 29%) as compared to solid tumors (all-grade: 37%; grade ≥ 3: 19%) (76). These findings argue for tumor-specific strategies to combat CRS. A host of additional potential therapeutics are available to reduce CRS in specific contexts. These include Anakinra, an IL-1 receptor antagonist that blocks the activity of IL-1 (57); TO-207, which inhibits the release of multiple cytokines such as IL-1β, IL-6, IL-8, IL-18, GM-CSF, MCP-1, and TNF-α (77); Ruxolitinib and Itacitinib, JAK/STAT inhibitors (78, 79); Dasatinib, a tyrosine kinase inhibitor (80); Piclidenoson and Namodenoson, A3 adenosine receptor agonists (81); and Lenzilumab, a GM-CSF neutralizing antibody (82).

## 4 Bispecific T cell engagers

Bispecific T cell engagers are fusion proteins that are generated by linking the targeting region of two antibodies: one arm binds to the tumor associated antigen and the other arm binds to T cells. When both arms are engaged, the bispecific T cell engager acts as a bridge and brings the T cell in close proximity to the tumor cell to promote T cell mediated cytotoxicity in an MHC independent manner. Bispecific T cell engagers have been reported to activate any CD3+ cell i.e. CD3+ CD4+ helper T cells, CD3+ CD8+ cytotoxic T cells, and CD3+ NKT cells to become effector cytotoxic cells (83).

The review from Tian et al. discusses different formats of bispecific T cell engagers (84). Based on their structure, bispecific T cell engagers can be divided into two major categories: a. IgG-like and b. non-IgG like. The primary approach for generating IgG-like bispecific T cell engagers involves recombining half-molecules from heterogeneous parental antibodies. Newer techniques for producing IgG-like bispecific T cell engagers involve modification of the heavy chain of the antibody to promote heterologous Fc matching. For example, the knobs-into-holes approach promotes heterodimerization between half-molecules through mutations on C_H_3 domains (85, 86). The Duobody platform controls Fab dynamic recombination exchange from different parental IgGs (87). To solve the problem of light chain mismatching, the CrossMab platform has been developed by Roche by exchanging the C_H_1 and the constant region of the light chain of one parental antibody. It contains two tumor antigen binders and one CD3 binder (88). The orthogonal Fab interface depends on electrostatic manipulation for interactions (89). The XmAb platform generated by Xencor allows the production of bispecific T cell engagers that are nearly identical to natural antibodies (90). The design of non-IgG like antibodies is relatively simple, and they have low immunogenicity due to the lack of an Fc fragment. The dual affinity retargeting antibody (DART) molecule consists of two engineered heterogeneous single chain variable fragments (scFv) which have exchanged their variable heavy chain regions (91). Other platforms include tetravalent antiparallel structure (TandAbs) with two binding sites each for CD3 and the tumor antigen (92), and BiTEs to connect two scFv (93).

As compared to non-IgG like bispecific T cell engagers, IgG based antibodies have longer half-lives as they are large in size and are more difficult for the kidney to clear. Also, the presence of Fc fragment allows neonatal Fc receptor (FcRn) mediated recycling and improved solubility and stability. Moreover, Fc domains of IgG-like bispecific T cell engagers can recruit NK cells and macrophages to induce antibody-dependent cell-mediated cytotoxicity (ADCC), antibody-dependent cell-mediated phagocytosis (ADCP) and complement-dependent cytotoxicity (CDC). On the other hand, the Fc-mediated immune functions can be non-desirable for IgG-like bispecific T cell engagers as they enhance antigen-independent cytokine release syndrome (CRS) due to crosslinking of CD3 and Fcγ receptors followed by nonspecific activation of immune cells. Also, the tumor permeability of IgG-like antibodies is lower due to high molecular weight (84, 94–96).

**Figure 2:**
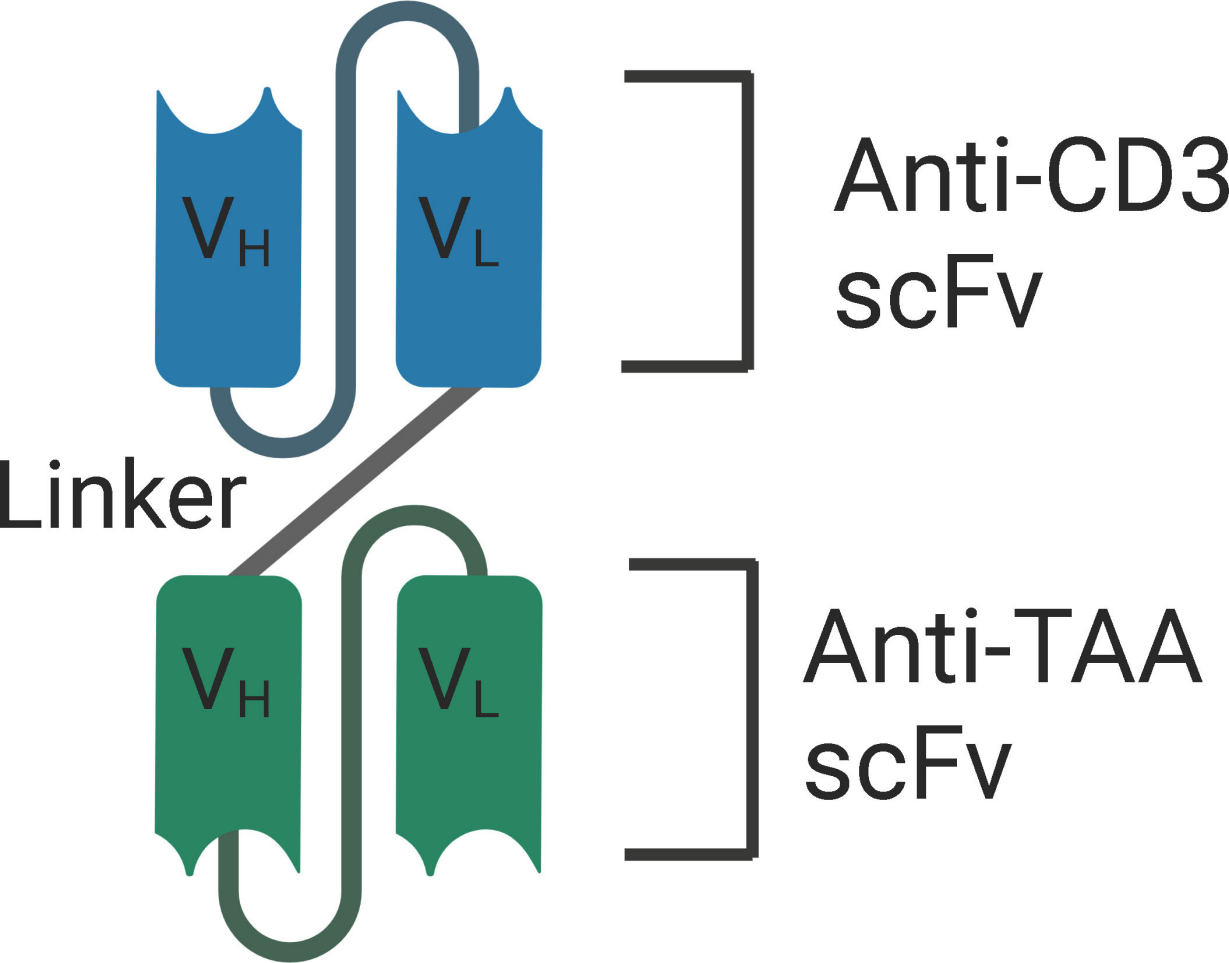
Structure of BiTE - BiTEs are fusion proteins that consist of two single-chain variable fragments (scFv) that are linked with a flexible linker. One fragment targets the CD3 subunit of the T cell receptor (TCR) complex, and the other fragment targets the tumor associated antigen. V_H_, Heavy chain variable regions. V_L_, Light chain variable regions. TAA, Tumor Associated Antigen.

Structurally, BiTEs are composed of two scFv, one fragment targets the CD3 subunit of the T cell receptor (TCR) complex, and the other fragment targets the tumor associated antigen. The two variable fragments are associated *via* a peptide linker (**Figure 2**). The length of the linker determines the flexibility and rotation of the molecule’s two targeting arms. BiTEs lack the Fc region of the two antibodies and are 55kDa and about 11 nm in length. Eliminating the Fc region of the antibodies might avoid the toxicities associated with the Fc effector functions, but also results in a lower half-life due to the elimination of neonatal Fc receptor (FcRn) mediated recycling (97–100). Importantly, simultaneous engagement of both the arms of BiTEs is required for T-cell activation and the release of cytokines – IFN-γ, TNF-α, IL-6, and IL-10. This enhances the specificity of BiTEs and minimizes the off-target adverse effects.

Several studies have shown that BiTEs are effective at a very low concentration of 10 to 100 pg/ml and very low effector cell to target cell ratios (<1:90). BiTEs induce T cell activation and cytotoxicity in the absence of T cell co-stimulation by IL-2 or anti-CD28 antibodies. This is attributed to the previously primed memory T cells. It is also suggested that the concurrent binding of BiTE with the tumor associated antigen and CD3 in the TCR complex forms a cytolytic synapse between the T cells and the tumor cells that mediate perforin and granzyme mediated killing of the tumor cells *via* caspase activation (84, 101, 102).

There are four bispecific T cell engagers that are currently approved by the FDA:

- Blincyto: CD19xCD3 BiTE for the treatment of children/adult B cell precursor acute lymphoblastic leukemia in patients with minimum residual disease or for the treatment of Philadelphia chromosome negative (Ph -) relapsed/refractory B-cell precursor acute lymphoblastic leukemia.
- Kimmtrak: gp100/HLA-A*02:01xCD3 bispecific for the treatment of unresectable or metastatic uveal melanoma.
- Tecvayli: BCMAxCD3 bispecific for the treatment of adult patients with relapsed or refractory multiple myeloma who have received at least four prior lines of therapy, including a proteasome inhibitor, an immunomodulatory agent, and an anti-CD38 monoclonal antibody.
- Lunsumio: CD20xCD3 bispecific for the treatment of adult patients with relapsed or refractory follicular lymphoma after two or more lines of systemic therapy.

**Table 2** describes the CRS incidence and manifestations reported with the use of these therapies.

**Table 2:** US-FDA approved Bispecific TCell Engagers.

Generic Name	Brand Name	Target Antigen	Targeted Disease	CRS Manifestation	%Incidence
Blinatumomab	Blincyto	CD19xCD3	Children and adult B-cell precursor acute lymphoblastic leukemia (ALL) in patients with minimum residual disease (MRD)	Fever, headache, nausea, asthenia, hypotension, increased alanine aminotransferase, increased aspartate aminotransferase, increased total bilirubin, and disseminated intravascular coagulation (DIC). The manifestations of CRS after treatment with BLINCYTO overlap with those of infusion reactions, capillary leak syndrome (CLS), and hemophagocytic histiocytosis/macrophage activation syndrome (MAS).	15% of patients with relapsed or refractory ALL and in 7% of patients with MRD-positive ALL (Common Terminology Criteria for Adverse Events - CTCAE grade).
			Philadelphia chromosome negative (Ph -) relapsed/refractory B-cell precursor acute lymphoblastic leukemia (ALL)		
Tebentafusp-tebn	Kimmtrak	gp100/HLA-A*02:01xCD3	Unresectable or metastatic uveal melanoma	Fever, hypotension, hypoxia, chills, nausea, vomiting, rash, elevated transaminases, fatigue, and headache	89% of patients experienced any grade CRS (2019 ASTCT grade) including 0.8% grade3-4 events and 1.2% discontnuations
Teclistamab-cqyv	Tecyavli	BCMAxCD3	Adult patients with relapsed or refractory multiple myeloma who have received at least four prior lines of therapy, including a proteasome inhibitor, an immunomodulatory agent, and an anti-CD38 monoclonal antibody.	Fever, hypoxia, chills, hypotension, sinus tachycardia, headache, and elevated liver enzymes (aspartate aminotransferase and alanine aminotransferase elevation).	72% of patients experienced any grade CRS (2019 ASTCT grade) Grade1 CRS (50% of patients), Grade2 CRS (21% of patients), Grade3 CRS (0.6% of patients), Recurrent CRS (33% of patients)
Mosunetuzumab	Lunsumio	CD20xCD3	Adult patients with relapsed or refractory follicular lymphoma after two or more lines of systemic therapy.	Fever of 100.4°F (38°C) or higher, headache, chills, confusion, low blood pressure, feeling anxious, fast or irregular heartbeat, dizziness or light-headedness, tiredness or weakness, nausea, difficulty breathing, vomiting	39% of patients experienced any grade CRS (2019 ASTCT grade) Grade 1 CRS (28% of patients) Grade 2 CRS (15% of patients) Grade 3 CRS (2% of patients) Grade 4 CRS (0.5% of patients) Recurrent CRS (11% of patients)

The information is based on the package insert of each FDA approved Bispecific TCell Engager therapy.

The onset of CRS for bispecific T cell engagers is earlier than for CAR-T cells due to the faster kinetics of cytokine release (55, 101). Leclercq et al. used an *in vitro* T-cell dependent cellular cytotoxicity (TDCC) model to understand the sequence of events in the CRS cascade. They co-cultured cancer cells expressing tumor associated antigens with either peripheral blood mononuclear cells (PBMCs), PBMCs without monocytes, or with total leukocytes in the presence of bispecific T cell engager. In line with the earlier reports, they found that bispecific T cell engager causes dose dependent CD4+ and CD8+ T cell activation and release of IL-2, IFN-γ, TNF-α, IL-6, IL-1β, and IL-8. Through intracellular cytokine staining by flow cytometry, they found that CD4+ and CD8+ T cells were positive for TNF-α and IFN-γ but not for IL-6 suggesting that T cells do not contribute to IL-6 release (103). Another study by *Li et al.* found that TNF-α derived from T cells contributed to the activation of myeloid cells, and monocytes and macrophages were the main mediators of IL-6 and IL-1β release. Blocking TNF-α prevented cytokine release without any impact on antitumor activity (104). The results concur with the findings from CAR-T studies (57, 58, 105) and with the findings from others who have highlighted the contribution of myeloid cells in mediating bispecific T cell engager related toxicity. Neutrophils were also reported to be activated upon bispecific T cell engager treatment and contributed to the release of IL-1β and other cytokines such as IL-8, IL-32, and MIP-1β (102, 103, 106).

As evident from **Tables 1**, **2**, CRS incidence with the use of bispecific T cell engagers is lower as compared to CAR-Ts. Due to its short half-life, the bispecific T cell engager treatment can be discontinued if needed without severe outcomes. Tumor burden and the initial dose of bispecific T cell engager have been identified as critical factors for CRS (107). Early intervention is therefore vital to prevent toxicity. The therapeutic strategy involves step dosing of the bispecific T cell engager, disease cytoreduction, and pretreatment with glucocorticoids. For low-moderate severity, CRS management includes dosing interruption and corticosteroid administration prior to resuming bispecific T cell engager dosing. The therapy is discontinued if severe CRS is manifested. Tocilizumab, the IL-6 inhibitor, may be used for CRS management either alone or in conjunction with corticosteroids and dosing interruption, especially for patients that are refractory to drug cessation and corticosteroids (10, 108–110).

For solid tumors, the challenge with bispecific T cell engager therapy as with CAR-T cells is the availability of tumor specific antigens. The antigens that are expressed at low levels in normal tissues might cause on-target off-tumor toxic adverse effects. Another potential issue is the suppressive tumor microenvironment created by the upregulation of immune checkpoints, immunosuppressive cells, and inhibitory cytokines. Other challenges include limited availability and penetration of intratumoral T cells. The baseline density of CD8+ cytotoxic T cells in the solid tumor is critical for efficacy. Clinicaltrials.gov reports eleven upcoming or active studies using bispecific T cell engagers for targeting various solid tumors. The tumor associated antigens for the bispecific T cell engagers in the trials include prostate-specific membrane antigen (PSMA) for metastatic castration-resistant prostate cancer; delta-like ligand3 (DLL3) for neuroendocrine prostate cancer, small cell lung cancer, and extensive stage-small cell lung cancer; MUC-17 for gastric, gastroesophageal junction, colorectal, and pancreatic cancers; PD-L1 for cervical cancer; six-transmembrane epithelial antigen of prostate 1 (STEAP1) for metastatic castration resistant prostate cancer; EGFRvIII for malignant glioma; and epidermal growth factor receptor (EGFR) for metastatic or locally advanced solid tumors (8, 101, 111, 112).

To enhance the efficacy and lower toxicity, novel classes of T cell engagers are being developed in addition to using a combinatorial approach. These include:

➢ Half-Life Extended (HLE) BiTE – Canonical BiTEs have a short half-life due to the lack of an Fc domain. Adding Fc domain to BiTE would prolong the half-life and eliminate the need for frequent infusions, as the binding of Fc with FcRn would lead to FcRn mediated recycling of the bispecific. HLE BiTEs targeting DLL3, FLT3, MUC17, and PSMA have been developed by Amgen and are in clinical trials for the treatment of gastric cancer, prostate cancer, colorectal cancer, pancreatic cancer, and small cell lung cancer (113, 114). However, work from Wang et al. showed that silencing the Fc domain of a bispecific T cell engager built on the IgG light chain-scFv platform by N297A ± K322A mutations enhanced T cell infiltration and anti-tumor efficacy in GD2+ neuroblastoma and HER2+ breast cancer xenograft models while eliminating ADCC related sequestration of T cells in the lungs or reticuloendothelial system (115).
➢ Simultaneous multiple interaction T cell engager (SMITE) – BiTEs cause T cell activation independent of CD28 activation. However, expression of CD80/CD86 on the cancer cells or co-administration of anti-CD28 *in vitro* enhanced the cytotoxic activity of BiTEs (116). This led to the concept of SMITE where two BiTEs can be administered – each of which binds to tumor associated antigen/checkpoint and either CD3 or CD28 (117).
➢ Probody bispecific T cell engager – Probody bispecific T cell engagers are prodrugs that are masked to eliminate antigen binding in healthy tissues and are activated by the proteases in the tumor microenvironment. In a pre-clinical setting, an EGFRxCD3 Probody bispecific T cell engager developed by CytomX showed promising results *in vitro* in terms of T cell activation, cytokine release, and cytotoxicity, and *in vivo* in terms of tumor growth inhibition and enhanced infiltration of T cells using a colon cancer xenograft co-engrafted with human PBMC in the NSG mouse host (118). To further refine this approach, Revitope’s Two GATE technology uses two bispecific antibodies with tumor specific protease – each targeting a different tumor associated antigen and a different half domain of the CD3 subunit; i.e. an active anti-CD3 complex is only formed when both halves of anti-CD3 come together to form a complex and activate T cells (119).
➢ Trispecific T cell engager – The trispecific approach involves targeting CD3+ T cells along with two different tumor associated antigens, or targeting CD3 and CD28 on the T cell along with the tumor associated antigen to enhance specificity, lower toxicity, and improve T cell activation (120, 121). Ongoing clinical studies include trispecifics that target immune checkpoints, three different tumor associated antigens, or two tumor associated antigens and human serum albumin for prolonging the half-life without the Fc fragment.

## 5 Immune checkpoint inhibitors

**Figure 3:**
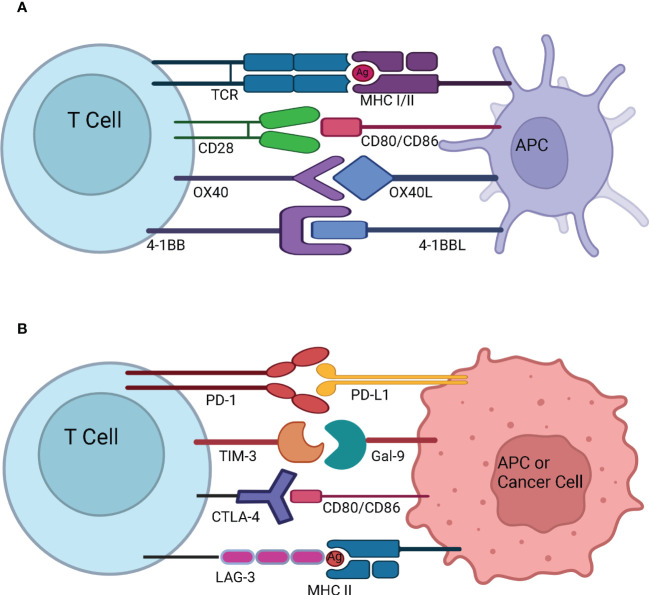
T-cell activation and immune checkpoints. **(A)** T-cell activation and stimulatory checkpoints - The first signal for T-cell activation is provided by the binding of T Cell Receptor (TCR) with the antigen (Ag) presented by the Antigen Presenting Cell (APC) in the context of Major Histocompatibility Complex (MHC). The second signal for T-cell activation comes from co-stimulation whereby CD28 on naïve T cells engages with CD80/CD86 on the APC. OX40 is present on the surface of activated T cells, and it interacts with OX40L present on the surface of APC. OX40-OX40L interaction promotes survival of T cells and enables generation and expansion of effector memory T cells. 4-1BB is an inducible costimulatory receptor expressed on the surface of activated T cells. It’s ligand 4-1BBL is expressed on the surface of APCs. The interaction of 4-1BB with 4-1BBL prevents activation induced cell death and results in proliferation and memory formation of CD8+ T cells **(B)** Immune Checkpoints - Programmed cell death protein-1 (PD-1) present on the surface of activated T cells interacts with Programmed death-ligand 1 (PD-L1) overexpressed on the surface of APC or cancer cell and suppresses the immune system. T-cell immunoglobulin and mucin-domain containing-3 (TIM-3) present on the surface of activated T cells interacts with Gal-9 on the surface of APC or cancer cell to dampen the T cell response. Cytotoxic T-Lymphocyte Associated Protein 4 (CTLA-4) expressed on the surface of activated T cells interacts with CD80/CD86 on the surface of APC or cancer cell and prevents further activation of T cells by blocking the interaction of CD28 with CD80/CD86. Lymphocyte-Activation Gene 3 (LAG-3) expressed on the surface of activated T cells binds to MHC II expressed by the cancer cell or APC. This prevents TCR – MHC II interaction and suppresses anti-cancer immunity.

Immune checkpoints are receptors expressed by the immune cells and act like an on/off switch to maintain the homeostatic balance of immune system suppression and upregulation. They prevent the overactivation of the immune system. However, cancer cells are known to evade the immune system by enhancing the immune checkpoint activation. Immune checkpoint inhibitors were developed to improve the anti-tumor response by the immune system. Currently, there are eight FDA approved immune checkpoint inhibitors for the treatment of various indications of cancer (**Table 3**). They have been effective in treating solid cancers. However, only a subset of patients respond to treatment. The underlying causes for patient specificity are currently under investigation, as are biomarkers of response. Checkpoint inhibitors may be useful for the recruitment of immune cells to the tumor microenvironment in combination with either bispecific T cell engagers or CAR-Ts to treat disease more effectively. **Figure 3** illustrates T-cell activation and immune checkpoints. Various classes of immune checkpoint inhibitors are described below.

**Table 3:** US-FDA approved Immune Checkpoint Inhibitors.

Generic Name	Brand Name	Immune checkpoint	Targeted Disease	CRS Manifestation and % Incidence
Pembrolizumab	Keytruda	PD-1	Advanced non-small cell lung cancer	No CRS related adverse events have been reported with the use of any checkpoint inhibitor in the package insert.
			Melanoma	
			Head and neck squamous cell cancer	
			High-risk non-muscle invasive bladder cancer	
			Advanced urothelial bladder cancer	
			Kidney cancer	
			Microsatellite instability-high cancer (MSI-H/dMMR)	
			Advanced MSI-H/dMMR colorectal cancer	
			High-risk early-stage triple negative breast cancer (TNBC)	
			Classical Hodgkin Lymphoma	
			Advanced gastric cancer	
			Advanced cervical cancer	
			Advanced MSI-H/dMMR endometrial cancer	
			Primary mediastinal B-cell lymphoma (PMBCL)	
			Advanced liver cancer (HCC)	
			Advanced Merkel cell carcinoma	
			Advanced esophageal cancer	
			Cutaneous squamous cell carcinoma (cSCC)	
Nivolumab	Opdivo	PD-1	Non-small cell lung cancer	
			Advanced renal cell carcinoma	
			Gastric, gastroesophageal junction, or esophageal cancer	
			Melanoma	
			Urothelial carcinoma	
			Unresectable malignant pleural mesothelioma	
			Recurrent or metastatic squamous cell carcinoma of the head and neck	
			Hepatocellular carcinoma (HCC)	
			Advanced MSI-H/dMMR colorectal cancer	
			Relapsed or progressed classical hodgkin lymphoma	
Cemiplimab	Libtayo	PD-1	Advanced non-small cell lung cancer	
			Locally advanced basal cell carcinoma	
			Advanced cutaneous squamous cell carcinoma	
Atezolizumab	Tecentriq	PD-L1	Adjuvant non-small cell lung cancer	
			Metastatic non-small cell lung cancer	
			Hepatocellular carcinoma	
			Melanoma	
			Urothelial carcinoma	
			Small cell lung cancer	
Avelumab	Bavencio	PD-L1	Locally advanced or metastatic urothelial carcinoma	
			Metastatic merkel cell carcinoma	
			Renal cell carcinoma (in combination with axitinib, a VEGFR inhibitor)	
Durvalumab	Imfinzi	PD-L1	Unresectable stage III non-small cell lung cancer	
			Extensive-stage small cell lung cancer	
			Advanced or metastatic biliary tract cancers	
Ipilimumab	Yervoy	CTLA4	Melanoma	
			Renal cell carcinoma	
			Hepatocellular carcinoma	
			Non-small cell lung cancer	
			Malignant pleural mesothelioma	
			Esophageal cancer	
Relatlimab (given in combination with Nivolumab)	Opdualag	LAG-3	Unresectable or metastatic melanoma	

The information is based on the package insert of each FDA approved checkpoint inhibitor. No CRS incidence has been reported in the package insert of any checkpoint inhibitor.

### 5.1 Programmed cell death protein-1 inhibitors and programmed death-ligand 1 inhibitors

PD-1 (CD279) is an inhibitory receptor that is expressed on the surface of activated T cells, B cells, NKT cells, NK cells, monocytes, myeloid derived suppressor cells, tumor associated macrophages, and dendritic cells (122–125). There are two known ligands for PD-1, PD-L1 (B7-H1) and programmed death-ligand 2 (PD-L2) (B7-DC). PD-L1 is expressed on T cells, B cells, antigen presenting cells, and in some non-lymphoid and normal tissues and malignant cancer cells.

PD-L2 expression was believed to be restricted to dendritic cells, macrophages, and bone-marrow derived mast cells. For this reason, PD-L1 has been the focus of many therapeutic strategies whereas the PD-1/PD-L2 interaction is not well studied in relation to cancer immunotherapy. However, recent studies have implicated the role of PD-L2 in various solid tumors (122, 126). In terms of PD-1/PD-L1, Han et al. have elaborately described the signaling pathways that impact their expression. These signaling pathways are frequently altered in tumor cells and include PI3 kinase/Akt, MAP kinase, JAK-STAT, WNT, NF-κB, and Hedgehog. The review also mentions micro RNAs and long non-coding RNAs that impact the expression of PD-1/PD-L1 (127).

The interaction of PD-1 with PD-L1 causes SH2-containing phosphatase 2 (SHP2) activation which impacts downstream signaling through PI3Kinase/Akt signaling that in turn lowers IL-2 and IFN-γ production and blocks T cell proliferation and survival. The engagement leads to T cell anergy and exhaustion. Also, the interaction lowers anti-apoptotic factors and upregulates pro-apoptotic factors leading to T cell apoptosis. The binding of PD-1 with PD-L1 increases E3 ubiquitin ligase Cbl-b expression which leads to T cell receptor (TCR) downregulation. Various studies have described the role of the PD-1/PD-L1 axis in the expansion and maintenance of immunosuppressive regulatory T cells (Tregs) and the conversion of CD4+ T cells to Tregs. The PD-1/PD-L1 interaction mediates resistance to CD8+ T cell mediated killing of tumor cells by forming a barrier between them. Literature also describes the role of PD-L1 as an anti-apoptotic receptor on cancer cells and preventing Fas ligation induced apoptosis of cancer cells (128, 129). Furthermore, signaling through PD-L1 protects the tumor cells from IFN-γ mediated cytotoxicity through STAT-3/Caspase7 dependent signaling (130).

PD-1 or PD-L1 inhibitors block the PD-1/PD-L1 interaction and thus relieve the brakes and enhance the response of T cells in the fight against cancer. PD-L1 expression on tumor cells serves as an important predictive biomarker for the use of PD-1 and PD-L1 inhibitors. However, based on the clinical outcomes of U.S. FDA approved checkpoint inhibitors, PD-L1 expression was predictive in only 28.9% of the cases, and was non-predictive in 53.3% of the cases across fifteen different types of cancer (131). The method used for the detection of PD-L1 expression, tumor microenvironment, and the mutational burden are important factors of consideration (132, 133). Employing a combinatorial approach using checkpoint inhibitors along with other targeted therapies is predicted to provide better clinical benefits.

### 5.2 Cytotoxic T-lymphocyte associated protein 4 inhibitor

**Figure 4:**
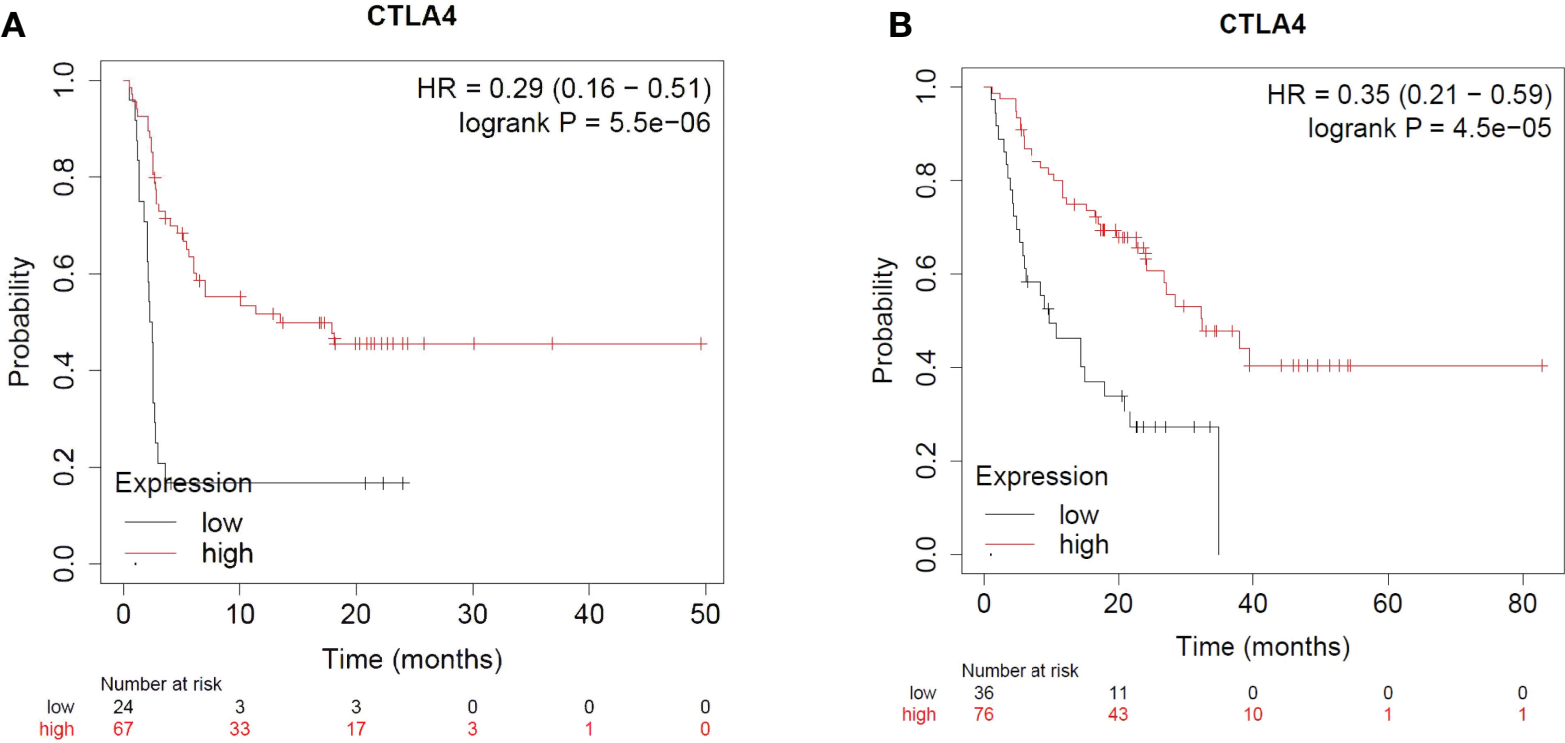
Impact of CTLA4 expression and Ipilimumab treatment in Melanoma. **(A)** Impact of CTLA4 expression and Ipilimumab treatment on Progression Free Survival in Melanoma. **(B)** Impact of CTLA4 expression and Ipilimumab treatment on Overall Survival in Melanoma.

CTLA-4 is upregulated when the antigen specific T cell receptor engages with a peptide presented in the context of an MHC. CTLA-4 has higher affinity and avidity for CD80/CD86 and competitively inhibits the binding of CD28 with CD80/CD86. Engagement of CTLA-4 with CD80/CD86 thus blocks the co-stimulation and attenuates T cell activation. The majority of cell-extrinsic suppression of CTLA-4 is mediated by Tregs likely by restricting the availability of CD80/CD86 for CD28 mediated co-stimulation of nearby effector T cells. Furthermore, CTLA-4 could also limit the availability of CD80/CD86 by mediating its transendocytosis from the antigen presenting cells (134). Ipilimumab is a monoclonal antibody that binds to CTLA-4 and prevents its interaction with CD80/CD86. CTLA-4 blockade has been shown to enhance T-cell activation and proliferation, including tumor infiltrating T-effector cells. Moreover, the blockade reduces Treg function which further enhances T-cell responsiveness and anti-tumor response. Ipilimumab is combined with Nivolumab for the treatment of certain cancers as the combination therapy has been found to be more effective as compared to the use of an individual checkpoint inhibitor (135–140). The combination therapy is more effective as it leads to the expansion of CD8 effector T cells as compared to monotherapy which causes the expansion of phenotypically exhausted CD8 T cells. Moreover, the reduction in Tregs in the tumors in combination therapy was higher than in the monotherapy groups (141). CTLA-4 expression – not just in the T cell lineage, but on tumors, have implications for immunotherapy and Ipilimumab response. Pistillo et al. showed that CTLA-4 expression in melanoma cells is correlated with Ipilimumab response and could possibly be used as a predictive biomarker of anti-CTLA-4 drug response (142). Using a transcriptomic dataset of patients treated with immune checkpoint inhibitors, it is evident that melanoma patients with higher CTLA-4 expression and receiving Ipilimumab therapy alone have significantly better progression free survival (PFS) and overall survival (OS) as compared to patients with low CTLA-4 expression (143) (**Figure 4**).

For the use of Ipilimumab alone or in combination with other checkpoint inhibitors across other indications of cancer (based on CTLA-4 expression), the sample size was not sufficient for meaningful analyses (143).

### 5.3 Lymphocyte-activation gene 3 inhibitor

LAG-3 is a co-inhibitory receptor that suppresses T cell activation and cytokine secretion. It is expressed on TILs - activated CD4+ and CD8+ T cells, Tregs, NK cells, γδT cells, NKT cells, and dendritic cells. LAG-3 binds with MHC-II with greater affinity than the CD4 T cell receptor which blocks TCR signaling and leads to immune suppression. LAG3-MHC-II interaction contributes to melanoma resistance to apoptosis. LAG-3 is expressed at a lower level on naïve CD8+ T cells, and its expression is increased on activated CD8+ T cells. Inhibiting LAG-3 is associated with an increase in the effector function of CD8+ T cells. LAG-3 promotes Treg differentiation and is essential for Treg suppressive function. Besides MHC-II, other ligands of LAG-3 include galectin-3 found on tumor and tumor stromal cells, liver sinusoidal endothelial cell lectin (LSECtin) which is expressed on liver and melanoma cells, and fibrogen-like protein 1 (FGL1) secreted from hepatocytes and tumor cells (144–146).

Higher expression of LAG-3 and infiltration of LAG-3+ cells in tumors are associated with poor prognosis, tumor progression, and unfavorable clinical outcomes in various indications of cancer. Several reports suggest that LAG-3 works concomitantly with PD-1/PD-L1 to enhance tumor induced tolerance and mediate antitumor immunity. For this reason, a combination treatment strategy of anti-LAG-3 along with anti-PD-1/anti-PD-L1 is recommended (147, 148). In March 2022, the U.S. FDA approved Opdualag – a fixed dose combination of LAG-3 inhibitor Relatlimab and PD-1 inhibitor Nivolumab for the treatment of patients with unresectable or metastatic melanoma. The combination therapy demonstrated better progression free survival in the clinical trial as compared to Nivolumab alone (149). As of 27^th^ February 2023, according to clinicaltrails.gov - there are 101 upcoming/ongoing clinical trials using anti-LAG3 either alone or in combination with other checkpoint inhibitors, targeted agents, or chemotherapeutic agents for various indications of cancer.

As demonstrated by **Table 3**, no CRS related adverse events have been reported with the use of any checkpoint inhibitor in the package insert. However, there have been several reports of rare cases of CRS with the use of immune checkpoint inhibitor therapy for various indications of cancer (7, 11–14, 150, 151). Combinatorial ICI therapy is associated with CRS as well. A case of fulminant CRS complicated by dermatomyositis was reported during the treatment of a renal cell carcinoma patient with Nivolumab and Ipilimumab (152). Another instance of Stage 4 CRS with Nivolumab and Ipilimumab combination therapy was observed in a patient with metastatic melanoma (153). Factors associated with high-grade CRS related to the use of immune checkpoint inhibitors are not well characterized. Although most patients recover, there are a few instances with fatal outcomes which warrant better clinical awareness for the optimal use of immune checkpoint inhibitors in cancer immunotherapy and associated CRS management.

## 6 *In vitro* and *in vivo* models of CRS evaluation

**Figure 5:**
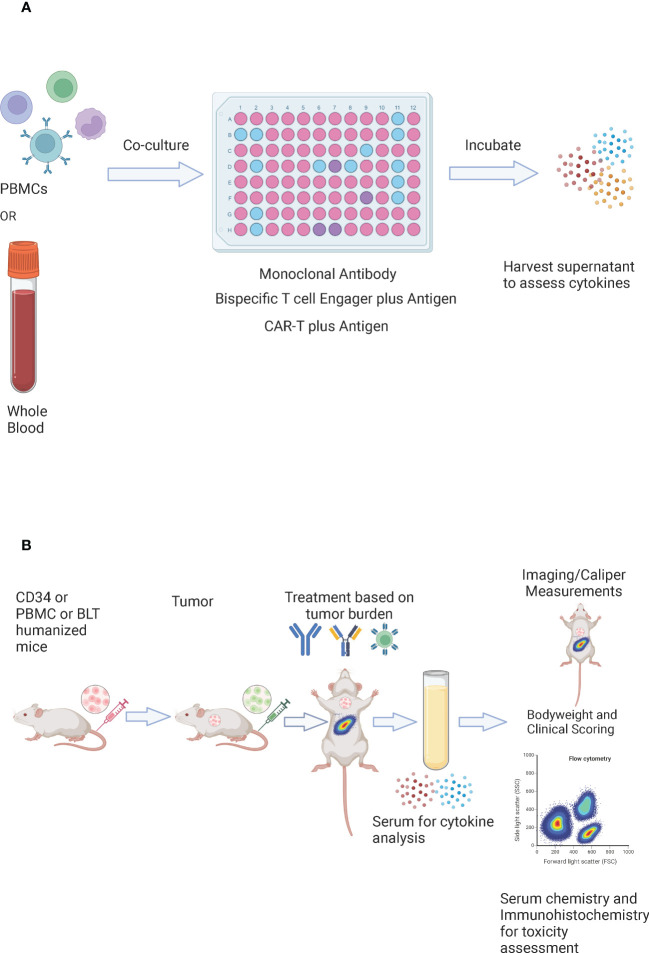
*In vitro* and *In vivo* evaluation of CRS. **(A)** *In-vitro* evaluation of CRS – whole blood or PBMCs are co-cultured with a monoclonal antibody, a bispecific T cell engager in the presence of an antigen, or CAR-T cells in the presence of an antigen. They are incubated for a specific amount of time and the supernatant is harvested for the assessment of cytokines. **(B)** *In vivo* evaluation of CRS – CD34, peripheral blood mononuclear cells (PBMC), or bone marrow-liver-thymus (BLT) humanized mice are injected with tumor cells (tumor cells may be luciferase tagged). The number and timing of tumor cells could be adjusted to reflect the appropriate tumor burden. The mice are randomized based on the tumor burden and are treated with CAR-T cells, a bispecific T cell engager, or a checkpoint inhibitor. Serum is harvested post dosing for cytokine analysis. Bodyweights and clinical scores are recorded post treatment. Imaging or caliper measurements could be performed to assess efficacy, and flow cytometry could be performed to assess the immune subsets. For toxicity assessment – serum chemistry could be performed, and tissues could be collected for immunohistochemistry.

*In-vitro* assessment of cytokine release involves co-culturing the investigational drug with PBMCs (**Figure 5A**). For monoclonal antibodies, the widely used platform requires culturing human PBMCs in the 96 well plate that is coated and air-dried with the antibody (solid phase method), in the presence of culture medium and donor plasma (2% v/v) at 37°C and 5% CO_2_ for a specific period of time. After the incubation period, the cell-conditioned medium is harvested for the assessment of cytokines *via* Enzyme-Linked Immunosorbent Assay (ELISA)/Mesoscale/Cytometric Bead Array (CBA)/Luminex platform (154). However, this approach is more prone to provide false-positive results as cytokines were detected with the antibodies that are known to have weak or rare/no CRS association (155). Other enhanced approaches include; high-density preculture of PBMCs for better assessment of monoclonal antibodies that induce cytokine release *via* Fcγ receptor (156), and autologous blood outgrowth endothelial cell (BOEC)-PBMC assay to mimic the effect of the interaction of monoclonal antibody with endothelial cells, avoid tissue mismatch and retain the disease phenotype of the patient (157). An alternative to using PBMCs is whole blood as it is more physiologically relevant. However, there are conflicting results about using whole blood for CRS risk assessment of different antibodies (158, 159). A study has demonstrated that whole blood assay is a better model for the detection of cytokine release by oligonucleotides that bind to toll-like receptors (160). For the CRS evaluation of bispecific antibodies, PBMCs are co-cultured with target cells expressing tumor associated antigen in the presence of a bispecific antibody for a specific period of time and the supernatant is harvested for the assessment of cytokines (103). To assess the CRS risk of effector CAR-T, the cells are co-cultured with target expressing cancer cells at a defined ratio in the presence of media for a specific amount of time and the cell culture supernatant is assessed for the presence of cytokines (161). Despite being rapid and cost-effective, the *in-vitro* models for CRS assessment do not provide details about the systemic immune response (vascular endothelium, tissue resident immune cells), off-target effects (cross-reactivity, unintended target activation), and about neurotoxicity, tissue damage, and organ failure. These shortcomings can be addressed through the *in-vivo* evaluation (162).

Non-human primate trials are considered as the benchmark for preclinical efficacy and drug safety assessment as they better mimic human physiology and disease progression. However, non-human primate work is time-intensive, expensive, and involves serious ethical concerns.

TGN1412 is a monoclonal antibody that is a superagonist of the costimulatory molecule CD28 expressed by human T cells. The preclinical work of TGN1412 in cultured human PBMC assays, rats, as well as cynomolgus monkeys failed to warn of the cytokine storm experienced by the human volunteers in the clinical trial. TGN1412 did not cause any observed adverse effect in cynomolgus monkeys receiving the monoclonal antibody at up to 500-fold higher doses than the human volunteers. A later study found that TGN1412 elicited a cytokine response *in vitro* only if it is appropriately presented to PBMCs e.g., by immobilization of TGN1412 onto the plastic surface, through coupling of TGN1412 *via* immobilized Fc-specific antibody or by co-culturing TGN1412 in the presence of an endothelial-like cell monolayer. No detectable cytokine release response was observed when TGN1412 was tested in the aqueous phase (154). These findings explain why *in vitro* testing of TGN1412 in the aqueous phase failed to evoke the cytokine release observed in humans. Further investigation revealed that the activation of CD4+ effector memory T cells by TGN1412 was the likely reason for the cytokine storm. Since the species used for the preclinical testing of TGN1412 lacked CD28 expression on CD4+ effector memory T cells, it failed to predict a cytokine storm that was observed in humans (163). This necessitates a more reliable preclinical model for drug development (164).

Humanized mouse models can serve as a valuable tool for the assessment and prediction of CRS *in vivo* (**Figure 5B**) (165). Several studies describe the use of immunodeficient mouse models involving engraftment with the human immune system to study CRS or other immune mediated adverse events (57, 58, 166–172). The models include- PBMC humanized NRG mice, bone marrow-liver-thymus (BLT) immune humanized mice, or PBMC humanized severe combined immunodeficiency (SCID) mice to evaluate monoclonal antibody mediated CRS (167). Giavridis et al. showed that severe CRS developed when SCID-beige mice were treated with human CD19-CAR-T cells in the context of a high intraperitoneal disease burden (57). Another report showed that CD34+ humanized SGM3 mice with high leukemia burden caused CRS upon CAR-T treatment (58). However, none of these models have emerged as a standard preclinical screening tool for immunotherapies. Recent work from James Keck’s group at The Jackson Laboratory describes a rapid, sensitive, and reliable *in vivo* pre-screening tool to predict the CRS toxicity of immunotherapeutic agents (173). This approach uses NOD‐*scid IL2rg^null^* (NSG) mouse platform and its variants – NSG-SGM3 that support better myeloid engraftment and NSG-MHC I/II DKO that exhibit delayed GvHD. NSG, NSG-SGM3, and NSG-MHC I/II DKO mice are irradiated (100 cGy) and injected with 20 million PBMCs. Six days post PBMC engraftment the mice are treated with either PBS, OKT3, or anti-CD28, and the serum is collected 6 hours post treatment for the assessment of various human cytokines – IFN-γ, IL-2, IL-4, IL-6, IL-10, and TNF-α. In response to OKT3 and anti-CD28, the model produced increased cytokine levels and behavioral and observational phenotypes consistent with human CRS. The model is independent of acute xenogeneic GvHD, effectively captures the variation in cytokine release between various PBMC donors, is reproducible between experiments using the same PBMC donor, and the magnitude of cytokine release is drug and dose dependent. A direct comparison with an *in vitro* assay using the same PBMC donors revealed that the *in vivo* assay was more sensitive for the detection of cytokine release from specific PBMC donors, highlighting the importance of the *in vivo* assay for risk assessment (173). The PBMC humanized mice can be co-engrafted with a luciferase tagged tumor allowing the model to concomitantly assess both the efficacy and safety profile of the therapeutic in the same assay period. Importantly, a combination of body weight, clinical assessment, and organ health can be used to assess toxicity as it relates to the overall magnitude and duration of cytokine release (174–176). The model can be used to predict the dose and donor specific safety profile of novel anti-cancer immunotherapeutics involving CAR-T cells, bispecific/trispecific T cell engagers, and immune checkpoint inhibitors (alone or in combination) (177). This model can be extremely valuable in drug discovery by de-risking during lead selection and in the clinical setting as the PBMCs from the patient can be used to provide a personalized prediction of whether the therapy will be safe and effective for that particular patient (178, 179).

## 7 Discussion

CRS is one of the most severe adverse events related to cancer immunotherapy. Although we have made great strides in CRS management through the use of Tocilizumab and corticosteroids, there is still room for improvement. Additional targets for therapeutic intervention in CRS need to be identified. The use of *in vivo* models to rapidly screen for the cytokine profile and efficacy would enable the assessment of the patient specific risk benefit ratio for immunotherapy. Approval of novel cytokine targeting agents that can diminish CRS mediated toxicity without affecting anti-tumor activity is critical for safety.

This review elucidates several novel classes of CAR-T cells and bispecific T cell engagers with modifications to enhance efficacy and minimize CRS toxicity. Other approaches to reduce CRS toxicity include the use of oncolytic viruses and dendritic cell vaccines for cancer immunotherapy. Moreover, to circumvent CRS associated with T cell activation, efforts are underway to employ other immune cells for CAR cell therapy like γδT cells, NKT cells, NK cells, neutrophils, monocytes, and macrophages (180). As the spectrum of secreted cytokines is different, these CAR therapies might be relatively safer. The other major benefit is that these therapies can be made available “Off-The-Shelf” in an allogeneic setting eliminating the manufacturing wait time. These approaches are less expensive, and healthy donor cells can be used for more potent therapy. Promising results of novel immunotherapies either alone or in combination with standard cancer therapies will pave the way for more safe and more effective cancer care.

## Author contributions

DS wrote the manuscript. BS and LS reviewed the manuscript and provided feedback for revision. All authors contributed to the article and approved the submitted version.
